# Management of end-stage heart failure: a perspective on the Arab Gulf states

**DOI:** 10.4103/0256-4947.57169

**Published:** 2009

**Authors:** Waleed Al Habeeb, Garrick C. Stewart, Gilbert H. Mudge

**Affiliations:** From the Division of Cardiovascular Medicine, Brigham and Women's Hospital, Harvard Medical School, Boston, Massachusetts, USA

## Abstract

The ever expanding epidemic of end-stage heart failure represents one of the greatest challenges of modern cardiovascular medicine. With medical treatments hampered by significant limitations, physicians caring for patients with advanced heart disease have turned to cardiac transplantation and durable mechanical circulatory assist devices as definitive therapies. These advanced therapeutic modalities are not widely available outside the United States and Europe, but nevertheless offer enormous potential for patients in the Arab Gulf suffering from end-stage heart failure. This review will discuss the management of end-stage heart failure in the Gulf States, with an emphasis on therapies best utilized within a framework of regional cooperation and coordination.

The management of patients with end-stage heart failure has emerged as one of the major challenges in cardiovascular medicine. There has been considerable progress in the understanding of heart failure pathophysiology leading to medical therapies that impact the prognosis and symptoms of chronic heart failure.^1^–^4^ Therapeutic options for end-stage failure, however, remain limited and include intravenous inotrope therapy, heart transplantation, or mechanical circulatory support.^5^,^6^ These advanced therapeutic modalities are available at select centers in the United States and Europe, but are not routinely considered in other global regions. The Arab Gulf is one such region. The primary barriers to widespread adoption of these advanced treatments are limited financial resources, lack of medical expertise, and cultural attitudes about organ procurement. The Arab Gulf is united by a common religion and culture and has a rapidly expanding medical expertise and proficiency. This review will discuss the management of end-stage heart failure in the Gulf States, with an emphasis on therapies that might be organized across national boundaries and closely coordinated by the Gulf's emerging tertiary care centers.

## Incidence of heart failure

The incidence of cardiovascular disease and heart failure is projected to increase substantially in the Arab Gulf States as the region completes an epidemiological transition fueled by socioeconomic change.^7^ Even as access to health technology increases, trends in urbanization, inactivity and receding infectious pandemics are allowing cardiovascular diseases to become the leading cause of morbidity and mortality. Hypertension is now estimated to affect more than one fourth of the Saudi population.^8^ The traditional high fiber, low fat diet has been replaced by a Western diet higher in fat. This change in dietary intake along with a more sedentary lifestyle has led to obesity in 35% of Saudi's as defined by a body mass index (BMI) ≥30 kg/m^2^ and diabetes mellitus in 23.7%.^9^–^13^ With atherosclerotic risk factors on the rise, coronary artery disease and ischemic cardiomyopathy will become more prevalent.^14^ Ischemic heart disease is already the leading cause of heart failure in Western Europe and the United States, countries that were the earliest to complete the epidemiologic transition.^15^,^16^ For example, heart failure currently accounts for over $35 billion in health care costs in the United States and remains the leading hospital discharge diagnosis in patients over the age of 65.^17^

It is estimated that 5-10% of heart failure patients have end-stage, refractory disease.^18^ These patients suffer from extreme exercise intolerance, debilitating dyspnea, often even at rest, and poor quality of life. The aggregate five-year survival rate of patients with heart failure is approximately 50 percent, whereas the one-year mortality rate of those with advanced disease may exceed 50 percent.^19^,^20^ This one-year mortality rate for New York Heart Association (NYHA) functional class IV heart failure exceeds that of HIV/AIDS and common malignancies, including breast, lung, and colon cancer.^17^ Caring for patients with the most advanced heart failure consumes over 60% of all health-care expenditures for patients with heart failure.^21^ This economic burden on the health care system is a consequence of frequent hospitalizations and the use of costly device therapies such as biventricular pacemakers and the implantable cardioverter defibrillators (ICDs).^22^–^26^ Heart failure patients are now less likely to suffer sudden cardiac death as a result of widespread use of neurohormonal antagonists and ICDs.^27^,^28^ These therapies have prolonged survival with heart failure leading to a larger proportion of patients in the later stages of this progressive disease who suffer the hemodynamic consequences of refractory fluid congestion and end-organ underperfusion. The increasing prevalence and severity of heart failure combined with the very poor quality of life and dismal prognosis mandate that other therapies be considered for heart failure patients living in of the Arab Gulf States.

## Medical therapies for advanced heart failure

The major advances in heart failure therapies have been primarily seen with systolic heart failure with a reduced ejection fraction. There is no debate that beta-adrenergic blockers, angiotensin converting enzyme (ACE) inhibitors, and angiotensin-receptor blockers (ARBs) offer an improved survival, and the frequency of their administration is emerging as a quality of care benchmark.^29^–^34^ Aldosterone antagonists have a clear role in post-infarction patients and symptomatic heart failure, although serum potassium and renal function must be carefully monitored.^35^–^38^ Hydralazine and nitrates continue to have a time-honored place in heart failure management, especially in those intolerant of ACE-inhibitors or ARBs or persistent renal compromise.^39^ Loop diuretic therapy has a central role for symptomatic relief but has not been shown to confer a survival benefit.^1^–^3^ Despite being available since the 18^th^ century, digitalis glycosides such as digoxin reduce hospitalizations for heart failure and provide modest symptomatic relief, but they do not prolonged survival.^40^,^41^

In patients with diastolic heart failure, the most effective therapies will include diuresis, control of blood pressure, and maintenance of sinus rhythm.^1^–^3^,^42^ There have been no studies demonstrating a robust mortality benefit of any agent in heart failure with a preserved ejection fraction even though such patients may constitute half of all patients with heart failure.^43^,^44^

Most medical therapies have been directed toward NYHA Functional Class II-III or AHA/ACC Stage C heart failure and have a limited role once the evolution of heart failure have moved to Class IV NYHA or Stage D (Tables 1 and 2). In fact, one definition of end-stage heart failure is an intolerance to or failure of conventional therapy.

**Table 1:** New York Heart Association functional class.

Class I – No limitation of physical activity. Ordinary physical activity does not cause undue fatigue, palpitation, or dyspnea (shortness of breath).
Class II – Slight limitation of physical activity. Comfortable at rest, but ordinary physical activity results in fatigue, palpitation, or dyspnea.
Class III – Marked limitation of physical activity. Comfortable at rest, but less than ordinary activity causes fatigue, palpitation, or dyspnea.
Class IV – Unable to carry out any physical activity without discomfort. Symptoms of cardiac insufficiency at rest. If any physical activity is undertaken, discomfort is increased.

**Table 2:** Stages of heart failure.

Stage A – At risk for heart failure but without structural heart disease or symptoms of heart failure
Stage B – Structural heart disease but without signs of symptoms of heart failure
Stage C – Structural heart disease with prior or current symptoms of heart failure
Stage D – Refractory heart failure requiring specialized interventional (e.g. mechanical circulatory assist devices, intravenous inotropes or vasodilators)

## Cardiac resynchronization therapy

Many heart failure patients with a low ejection fraction often also have interventricular conduction delay, as manifest by a widened QRS complex (>120 msec) on electrocardiogram from either a bundle branch block or nonspecific conduction delay. This delay creates dyssynchronous contraction of the left ventricle and can contribute to remodeling, mitral regurgitation and symptoms of congestion.^23^ Class III-IV Heart failure patients with reduced ejection fraction and conduction delay (QRS width >120-150 msec) may benefit from cardiac resynchronization therapy (CRT), also known as biventricular pacing.^25^ Pacemaker leads are placed in the right ventricular apex and the lateral free wall of the left ventricular via the coronary sinus to “resynchronize” contraction of the left ventricular. This may lead to reverse remodeling, decrease mitral regurgitation and a mortality benefit in select patients.^26^,^45^ Not every patient will respond to CRT. Prospectively identifying responders and nonresponders is an active area of research.

## Heart failure management programs

The primary goals in the management of end-stage heart failure are to reduce hospitalizations and to improve quality of life. Disease management programs have been shown to be effective in this regard, providing a structured continuity between the hospital discharge and the home setting.^46^,^47^ Pre-discharge education is central to its success. A team of providers that includes physicians, nurses, nurse practitioners, physicians assistances and pharmicists, working with established guidelines, can provide meaningful interventions when there is frequent communication with outpatients.^48^ For example, daily weights can be monitoring to guide adjustments of diuretic dosage and home blood pressure readings can be used to adjust afterload reduction.^49^ Implantable technology for daily estimation of left heart filling pressures can provide additional hemodynamic insights by telemonitoring.^50^ Heart failure management programs are most effective when established as part of a larger commitment to advanced heart failure care, including transplant or mechanical support.

## Management of end-stage heart failure

### Inotropic therapy

Many patients with refractory congestive symptoms and low cardiac output despite conventional therapy will benefit from infusion of a positive inotrope.^51^ Most commonly performed as an inpatient strategy, select patients can be transitioned to outpatient therapy infusion therapy once safe administration has been demonstrated.^52^ Though intravenous inotropes such as dobutamine (a beta-adrenergic receptor agonist) or milrinone (a phosphodiesterase-3 inhibitor) provide short-term improvements in congestive symptoms and cardiac output, this comes at the price of increased risk of ventricular tachycardia and direct cardiac myocyte toxicity.^53^,^54^ Prophylactic ICD are routinely placed to protect patients on chronic inotropic infusions from malignant tachyarrhythmias.^51^ Continuous outpatient inotropic therapy may be administrated through a tunneled central venous catheter and can help to maintain end-organ perfusion in patients awaiting heart transplantation.

### Heart transplantation

Orthotopic cardiac transplantation remains the definitive therapy for terminal heart failure.^55^ Surgical techniques can be acquired by many tertiary care centers. Recipient criteria have been well-established and immune suppression protocols have been standardized. Long-term post-transplantation care is managed by an integrated team of cardiologists, nurses, nurse practitioners, physicians assistants and pharmacists. After cardiac transplantation patients can achieve an acceptable 5-year survival of 70%, a 10-year survival of 60%, and markedly improved quality of life.^56^ Despite these impressive results in select patients, the ultimate potential for cardiac transplantation to stem the tide of the rising heart failure epidemic remains limited. Even in the United States, donor organ availability has remained static even as the waiting list for heart transplant grows.^57^–^59^

The primary barrier to cardiac transplantation in the Gulf States is the ad hoc organization of donor supply. While a limited number of centers have been able to perform the intermittent transplant, no program in the Gulf has achieved sufficient experience that would meet program certification goals of national regulatory bodies. Saudi Arabia is the only country within the Gulf region with a solid organ transplant organization, the Saudi Center for Organ Transplantation (SCOT), and King Faisal Specialist Hospital in Riyadh is the only heart transplant center. In 2008, the last year for which data is available, there were 19 cardiac transplantations in SCOT, preceded by 12 transplants in the year prior.^60^ To put this in perspective, the United States Center for Medicare Services requires advanced heart failure programs to perform 12 transplants per year for 3 years to secure certification. The absence of a well-organized solid organ procurement apparatus in the Gulf States has prevented the expansion of cardiac transplantation as a viable modality of therapy for select end-stage heart failure patients.

Sadly, mortality is rapidly rising in the Gulf states from motor vehicle collisions as well as cerebral vascular accidents from poorly controlled hypertension—the most common conditions causes brain death while preserving the potential for organ donation. While automobile accidents and strokes present a significant challenge to public health authorities, they also represent an opportunity to coordinate donor management, procurement and distribution. The Gulf Cooperative Countries (GCC) have already established political, travel and trade alliances. Established in 1981, the six GCC countries include Saudi Arabia, United Arab Emirates, Kuwait, Oman, Qatar and Bahrain with a population base approaching 41 million and a unifying culture. Organization of solid organ transplantation dovetails with the GCC charter which specifically outlines a goal of integration and inter-connection between members states in all fields to encourage cooperation and joint ventures.

Many existing organ procurement organizations cross political boundaries to serve the collective transplant need. In the United States, the New England Organ Bank represents 6 states and Bermuda, with a population base of 11.2 million. The Eurotransplant Interntational Foundation serves the transplant needs of Austria, Belgium, Germany, Luxembourg, the Netherlands, Slovenia and Croatia, with a population of 124.5 million and coordinated 6,600 solid organ donations. In order to address the current and future needs of all solid organ transplantation for the GCC, a coordinated transnational organ procurement organization representing the interests of the populations of all the Gulf States must be considered.

It is beyond the scope of this article to discuss the many religious and cultural issues surrounding solid organ transplantation. Organ donation, harvest and transplantation are a relatively new cultural concept in the Islamic and Arabic world and subject to a variety of interpretations. Renal transplantation, given its longer history, has wider acceptance than other solid organ programs.^61^–^63^ The Islamic medical community may have different interpretations than the general community, and at the present time, many medical centers follow the dictum that organ donation can be religiously rewarded—*And if any one saved a life, it would be as if he saved the life of the whole people. (Al-Maeda, Chapter 5, Verse 32)^64^*

### Ventricular assist devices

Given the immediate limitations to cardiac transplantation in the Gulf States and ultimate limitations of donor supply, ventricular assist devices (VAD) may be the most reasonable option for long-term hemodynamic support.^6^,^65^,^66^ (Tables 3 and 4) VAD technology has rapidly advanced, and such may now be considered as a reasonable therapy for both transplant eligible patients and those excluded due to conventional co-morbidities.^67^ VADs provide mechanical circulatory assistance after being placed in parallel to the patient's own heart which is left in its native anatomic position, unlike the total artifical heart or orthotopic transplantation.^68^ VADs are broadly categorized as pulsatile pumps or continuous flow rotary devices. Pulsatile assist devices were the first to be developed and the first approved for destination therapy (Heartmate XVE) after the landmark REMATCH trial.^69^ New technology includes the axial or continuous flow devices.^70^ These next generation VADs have a lower profile, making them easier to implant even in smaller patients, and, offer quiet, vibration free operation. These continuous flow pumps may also be easier to manage by the patient and their family, and seem to have extended durability compared to the pulsatile technology. By late 2008, there was evidence that the axial flow technologies were emerging as superior technology to the pulsatile devices, and it is anticipated that they might be the technology of choice in the near future. Each of these devices can be managed in the outpatient setting and permit full ambulatory capability.

**Table 3:** Indications for ventricular assist device implantation.

NYHA functional class IV symptoms
Life expectancy <2 years
Not a heart transplantation candidate
Failure to respond to optimal medical management for 60 of the last 90 days
Left ventricular ejection fraction ≤25%
Refractory cardiogenic shock or cardiac failure‡
Peak oxygen consumption ≤12 ml/kg/min with cardiac limitation
Continued need for intravenous inotropic therapy limited by symptomatic hypotension, decreasing renal function or worsening pulmonary congestion
Recurrent symptomatic sustained ventricular tachycardia or ventricular fibrillation in the presence of an untreatable arrhythmogenic pathologic substrate
Body surface area >1.5 m^2^±

NYHA: New York Heart Association;

‡ Cardiogenic shock may be seen following acute myocardial infarction or cardiac surgery; Implantation should only be considered in patients without potential for recovery;

± Smaller individuals may be fitted with available paracorporeal, small-sized pulsatile or newer axial-flow devices.

**Table 4:** Contraindications to ventricular assist device implantation.

Relative contraindications
Age>65 years, unless minimal or no other clinical risk factors
Chronic kidney disease with serum creatinine level >3.0 mg/dL
Severe chronic malnutrition (BMI<21 kg/m^2^ in males and <19 kg/m^2^ in females)
Morbid obesity (BMI>40 kg/m^2^)
Mechanical ventilation
Severe mitral stenosis, moderate to severe aortic insufficiency, or uncorrectable mitral regurgitation
Absolute contraindications
Reversible, transient causes of heart failure
Recent or evolving stroke
Neurologic deficits impairing the ability to manage device Coexisting terminal condition (e.g., metastatic cancer, cirrhosis)
Abdominal aortic aneurysm ≥5 cm
Biventricular failure in patients older than 65 years
Active systemic infection or major chronic risk for infection
Fixed pulmonary or portal hypertension
Severe pulmonary dysfunction (e.g., FEV_1_<1 liter)
Impending renal or hepatic failure
Multiple organ system failure
Inability to tolerate anticoagulation
Heparin induced thrombocytopenia
Significant underlying psychiatric illness or lack of social support that may impair ability to maintain and operate VAD

BMI: body mass index; COPD: chronic obstructive pulmonary disease; FEV_1_: forced expiratory volume in 1 second. Adapted from Wilson, et al.^81^

The clear survival advantage and quality of life for patients receiving VADs compared to medical therapy now justifies their use for circulatory support well beyond the transplant setting. In REMATCH, the implantation of a left-sided VAD was associated with a relative reduction in the risk of death of 48 percent during the entire follow-up period of 4 years and an absolute reduction in the mortality rate of 27 percent at one year.^69^,^71^ Quality of life with a VAD is substantially better than that experienced by patients just before VAD support. Many patients move from NYHA Class IV to Class I-II. These results portend an acceptable quality of life for the long-term use of VADs as an alternative to medical therapy for patients in need of cardiac transplantation but who will not receive it.^72^

Prior to VAD implantation, all patients should be evaluated at cardiac centers with specific expertise in advanced heart failure.^73^ Oftentimes patients benefit from more aggressive and expert management of their heart failure, including a restructuring of their medical regimen.^74^ If symptoms remain refractory to standard therapy, patients may be considered for VAD implanatation in one of three categories: 1) “Bridge to Recovery” for patients who are not expected to need long term support; 2) “Bridge to Transplantation” for patients suitable for cardiac transplant but who are too ill to wait until an organ becomes available; 3) “Destination Therapy” for patients needing long-term support but who are ineligible for cardiac transplantation by conventional criteria.^75^ The distinction between “bridge to transplant” and bridge to destination” may be too arbitrary. There will be inevitable “bridge to transplant” patients that develop complications precluding successful transplant, and there will be inevitable “bridge to destination” patients who improve sufficiently to be able to be transplanted without complication. Hence, “bridge to decision” is perhaps a more honest representation of options to many patients and their families.

The ample costs associated with VAD implantation are contingent on the type of device used, length of hospital stay, country-specific health economics, and the nature of the underlying disease.^76^–^78^ The mean total costs for VAD implantation is US $210 132 over an average period of 9.5 months.^79^ This does not meet current thresholds for cost-effectiveness, as expensive technologies can be cost effective while inexpensive ones can be cost ineffective. In the Gulf States, most of the health expenses are carried by the central governments and providing such expensive therapy may be considered a burden unless proven cost-effective. The ultimate comparison will balance the costs of treating heart failure with the incalculable costs of death and disability.

Any cost-effectiveness analysis must also take into account the rapid evolution of technology used. Since the axial flow devices are smaller, simpler to use, and more durable, it can be anticipated that they will also be more cost effective. In addition, the cost of a new medical technology usually declines as it becomes more widespread and management issues standardized. Continued improvements to the next generation of devices can be anticipated to extend durability and reduce complication rates.^80^

## Conclusions

The management of heart failure is both a challenge and an opportunity for the health care systems of the Gulf region. Advanced therapies for terminal heart failure remain elusive. While cardiac transplantation remains a potential option, there are logistical limitations in the Arabic world at the present time that limit widespread applicability. The new technologies of mechanical circulatory assistance may be a better option for end-stage patients, and requires thoughtful analysis and candid discussion about the priorities of health care resource allocation. Heart failure patients in the Gulf are deserving of these advanced therapies that will improve the quality and length of their lives.
